# Rare case of lymphomatoid papulosis type E^☆^

**DOI:** 10.1016/j.abd.2025.501133

**Published:** 2025-06-18

**Authors:** Máté Attila Bognár, Csaba Gyömörei, Zsuzsanna Lengyel

**Affiliations:** a Department of Dermatology, Venereology and Oncodermatology, University of Pécs, Pécs, Hungary; b Department of Pathology, University of Pécs, Pécs, Hungary

Dear Editor,

Lymphomatoid Papulosis (LyP) is a chronic, self-limited, usually relapsing papulonodular skin condition. Despite belonging to CD30-positive cutaneous lymphoproliferative disorders and malignancies, most cases follow a benign course, however, patients possess an increased risk of developing other hematological malignancies. According to the 2018 update of the WHO-EORTC classification for primary cutaneous lymphomas histologically, six different subtypes are differentiated: LyP type A‒E and a rare variant including the presence of DUSP22-IRF4 rearrangement.^1^ LyP type E with papules and plaques developing into eschar-like necrotic lesions is responsible for less than 5% of LyP cases, histologically characterized by angiodestructive, angioinvasive pleomorphic CD30+ T-cells.^2^, ^3^

A 71-year-old male patient with an extensive history of non-melanoma skin cancers was referred to our clinic, showing erythematous papulonodular lesions, progressing into ulcerated, necrotic lesions symmetrically on the lower extremities (Fig. 1). As the first symptoms appeared, levofloxacin therapy was administered with little to no result. The location and appearance of skin lesions made differential diagnosis challenging; necrotizing vasculitis, ecthyma gangrenosum and hematological malignancies were the most probable conditions. An investigation of the patient’s history ruled out new medications and infections previous to symptoms. Laboratory test results were normal, cryoglobulin level was not detectable. The immunological panel showed no abnormalities, viral serology was positive for CMV and EBV IgG. An X-ray of the chest and ultrasound of the abdomen did not find pathological anomalies. The excision specimen depicted epidermal ulceration and dermal-based angiodestructive medium-sized, to large-sized atypical lymphocytic infiltrate without extension into the subcutaneous fat. Mitotic figures were not infrequent. In addition to fibrin deposition, thrombosis was detected in some of the affected vessels. Tumor cells were extensively immunoreactive for CD3 and CD30 but did not stain positively for CD20. The neoplastic cells also expressed CD8, CD4, CD56, TIA-1 and Mum-1 (Fig. 2). Monoclonal TCR-g gene rearrangement was detected by PCR. The main histological differential diagnosis of an angiocentric/angiodestructive cutaneous lymphoid infiltrate includes lymphomatoid papulosis type E, extranodal NK/T cell lymphoma nasal type, hydroa vacciniforme-like lymphoma, lymphomatoid granulomatosis and cutaneous gamma/delta T-cell lymphoma. To effectively rule out systemic hematological malignancies, flow cytometry and peripheral blood smear were performed. Leukocytes and red blood cells showed no abnormalities, lymphocytes’ CD4/CD8 ratio was decreased (0.8). Physical examinations showed rapid progression; papulonodular lesions extensively became ulcerated leaving behind slowly healing necrotic lesions (Fig. 3). Upper body regions were not affected, nor were enlarged lymph nodes found. The patient did not report B symptoms. Due to rapid progression, 25 mg acitretin was administered, local disinfectant and a potent topical corticosteroid were used. Following three months of continuously developing new nodules and persisting necrotic ulcers, the patient reported fewer and fewer new lesions in which the ulcers were gradually healing. Regular follow-ups showed regression of all skin lesions by six months, adverse events were not reported with acitretin and frequent lab tests showed normal results. At the time of publishing, the patient has been followed up regularly for four years and showed no sign of relapse.

**Figure 1:**
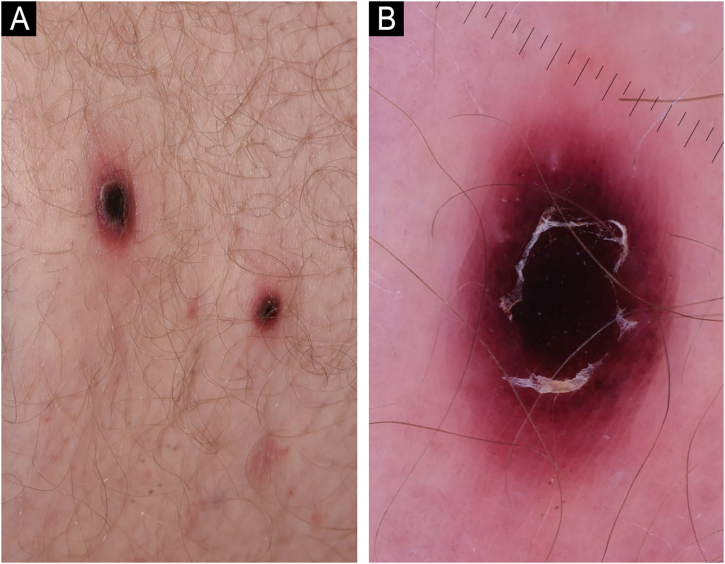
Erythematous papulonodular lesions (A) progressing into necrotic ulcers (B).

**Figure 2:**
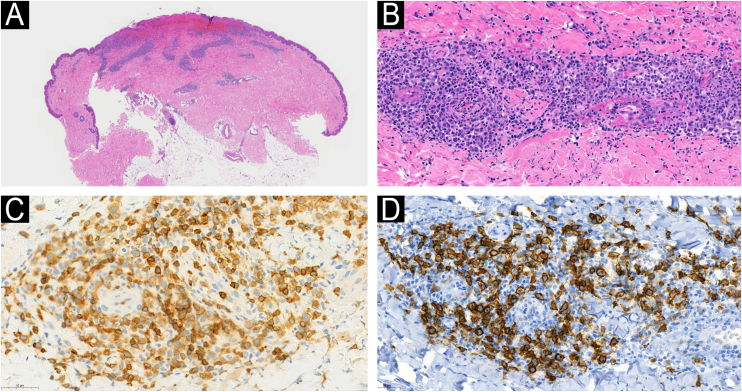
Low-power view showing epidermal ulceration and a dermal-based angiocentric lymphocytic infiltrate (Hematoxylin & eosin, original magnification 15×) (A). High-power image showing an angiodestructive medium-sized, to large-sized atypical lymphocytic infiltrate (Hematoxylin & eosin, original magnification 400×) (B). The tumor cells were extensively immunoreactive for CD3 (C) and CD30 (D) (CD3 and CD30 immunostain, original magnification 384× and 279×).

**Figure 3:**
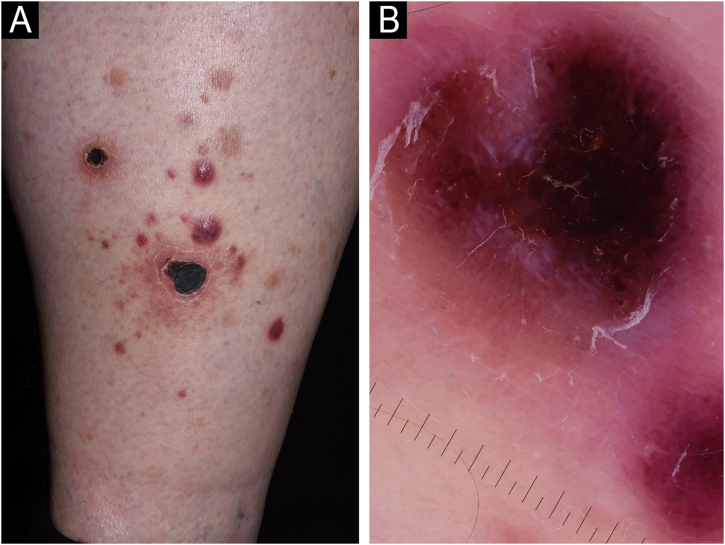
Extensive ulceration and necrosis of the lesions (A‒B).

Most LyP cases are self-healing, however, therapeutic intervention is recommended only in progressive and severe cases. Distinctively, the “wait and see” strategy is preferred among asymptomatic patients. Several treatment modalities are available, including local and systemic immunomodulators with or without the use of UVB. In the case of severe symptoms, methotrexate 2.5‒20 mg/week is the standardized, opted therapeutic choice preventing the formation of new lesions.^4^ Due to the significantly increased risk of non-melanoma and melanoma skin cancer in methotrexate-treated patients^5^ and our patient’s decades-long skin cancer history, a different treatment strategy was chosen. Acitretin, known for its anti-tumor proliferation effect, especially in UV-induced carcinogenesis and successful use in cutaneous CD30-positive anaplastic large cell lymphoma,^6^ was our first therapeutic choice deemed suitable in the treatment of our patient. Despite LyP being benign in nature, a majority of cases typically experience relapsing within months to years, while 10%‒40% of patients developing other hematological malignancies or transformation into CD30-positive Anaplastic Large Cell Lymphoma (ALCL), a regular follow-up is recommended even following total remission. Methotrexate, being the most effective systemic treatment regarding LyP, bears potential side effects in which patients’ intolerance can limit its use. In our case, we successfully administered acitretin with complete remission. After ceasing treatment, the patient reported no relapses over the four-year follow-up period.

## Financial support

None declared.

## Authors’ contributions

Máté Attila Bognár: Drafting and editing of the manuscript; collection, analysis, and interpretation of data; preparing the manuscript; critical review of the literature.

Csaba Gyömörei: Analysis and interpretation of data; intellectual participation in the propaedeutic and/or therapeutic conduct of the studied case; critical, approval of the final version of the manuscript.

Zsuzsanna Lengyel: Design and planning of the study, analysis and interpretation of data; intellectual participation in the propaedeutic and/or therapeutic conduct of the studied case; critical review of the literature; critical review of the manuscript; approval of the final version of the manuscript.

## Conflicts of interest

None declared.
